# Supplementary material for: Hydrogel crosslinking modulates macrophages, fibroblasts, and their communication, during wound healing
Source: Nat Commun. 2024 Aug 9;15:6820. doi: 10.1038/s41467-024-50072-y (PMC11315930; doi:10.1038/s41467-024-50072-y)
Supplement: Supplementary file 1 — Supplementary Information [file 41467_2024_50072_MOESM1_ESM.pdf]

Supplementary Information for:

Hydrogel crosslinking modulates macrophages, fibroblasts, and their communication, during wound healing

Sergei Butenko[1,2,16], Raji R. Nagalla[1,16], Christian F. Guerrero-Juarez[3], Francesco Palomba[4], Li-Mor David[1], Ronald Q. Nguyen[1], Denise Gay[2], Axel A. Almet[5-7], Michelle A. Digman[1,4], Qing Nie[5-7], Philip O. Scumpia[8-10], Maksim V. Plikus[2,6,11] and Wendy F. Liu[1,12-15, *]

1. Department of Biomedical Engineering, University of California Irvine, Irvine, CA, USA
2. Department of Developmental and Cell Biology, University of California, Irvine, Irvine, CA, USA
3. Carle Illinois College of Medicine, University of Illinois at Urbana-Champaign, IL, USA
4. Laboratory of Fluorescence Dynamics, The Henry Samueli School of Engineering, University of California, Irvine, CA, USA
5. Center for Complex Biological Systems, University of California Irvine, Irvine, CA, USA
6. NSF-Simons Center for Multiscale Cell Fate Research, University of California, Irvine, Irvine, CA, USA
7. Department of Mathematics, University of California, Irvine, Irvine, CA, USA
8. Division of Dermatology, Department of Medicine, David Geffen School of Medicine, University of California, Los Angeles, Los Angeles, CA, USA
9. Jonsson Comprehensive Cancer Center, David Geffen School of Medicine, University of California, Los Angeles, Los Angeles, CA, USA
10. Department of Dermatology, Veterans Administration Greater Los Angeles Healthcare System, Los Angeles, CA, USA
11. Sue and Bill Gross Stem Cell Research Center, University of California, Irvine, Irvine, CA, USA
12. UCI Edwards Lifesciences Foundation Cardiovascular Innovation and Research Center, University of California Irvine, Irvine, CA
13. Institute for Immunology, University of California, Irvine, Irvine, CA, USA
14. Molecular Biology and Biochemistry, University of California, Irvine, Irvine, CA, USA
15. Department of Chemical and Biomolecular Engineering, University of California Irvine, Irvine, CA, USA
16. These authors contributed equally to this work.

*Corresponding author.

Supplementary figures

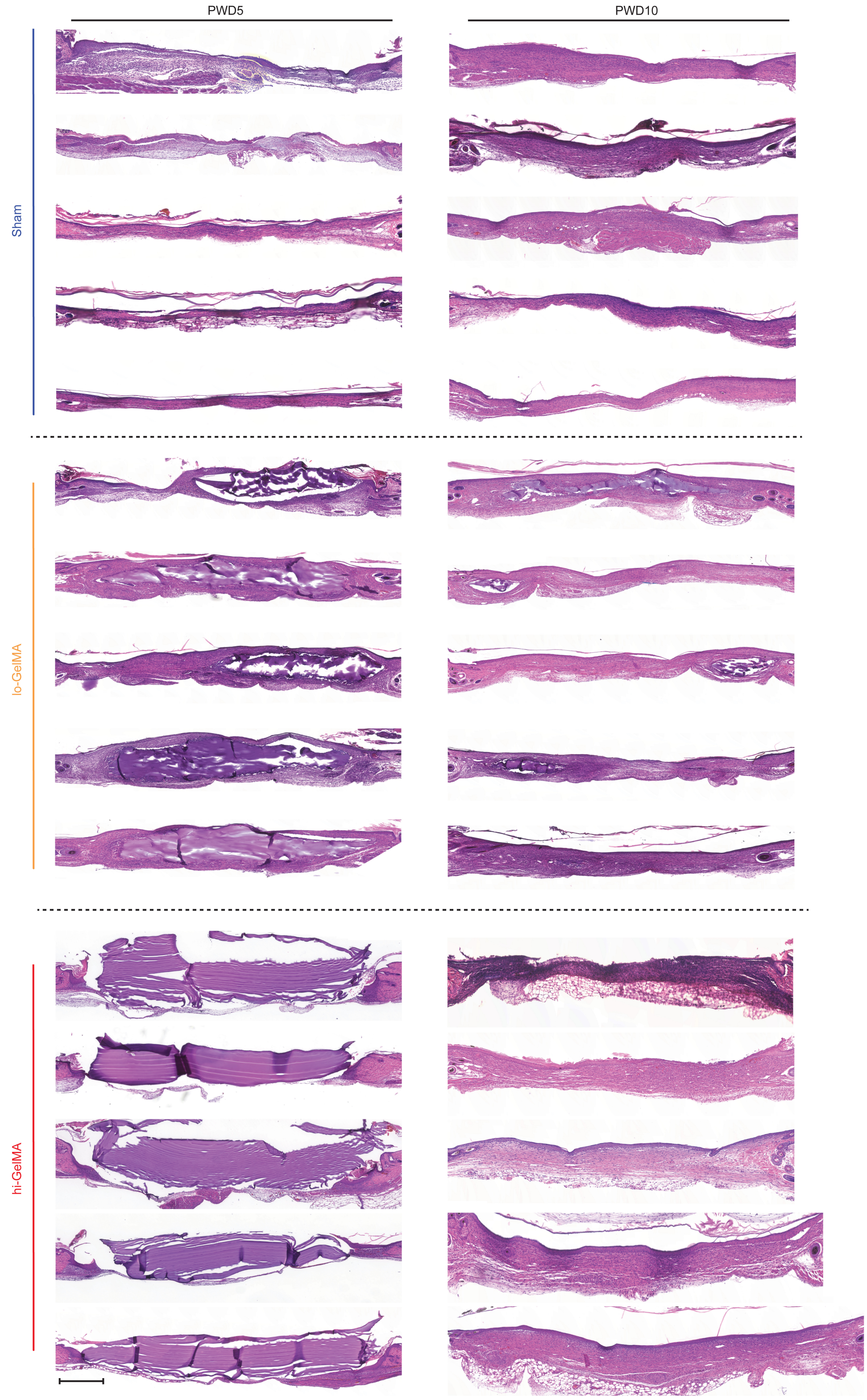

Supplementary Fig. 1. Additional H&E staining of PWD5 and 10 wound sections

Images of H&E stained PWD5 and 10 showing sham wounds along with GelMA treated wounds with incorporation and degradation of lo-GelMA vs. inflammation with cell aggregation and extrusion of hi-GelMA. Scale bar: 200 μm.

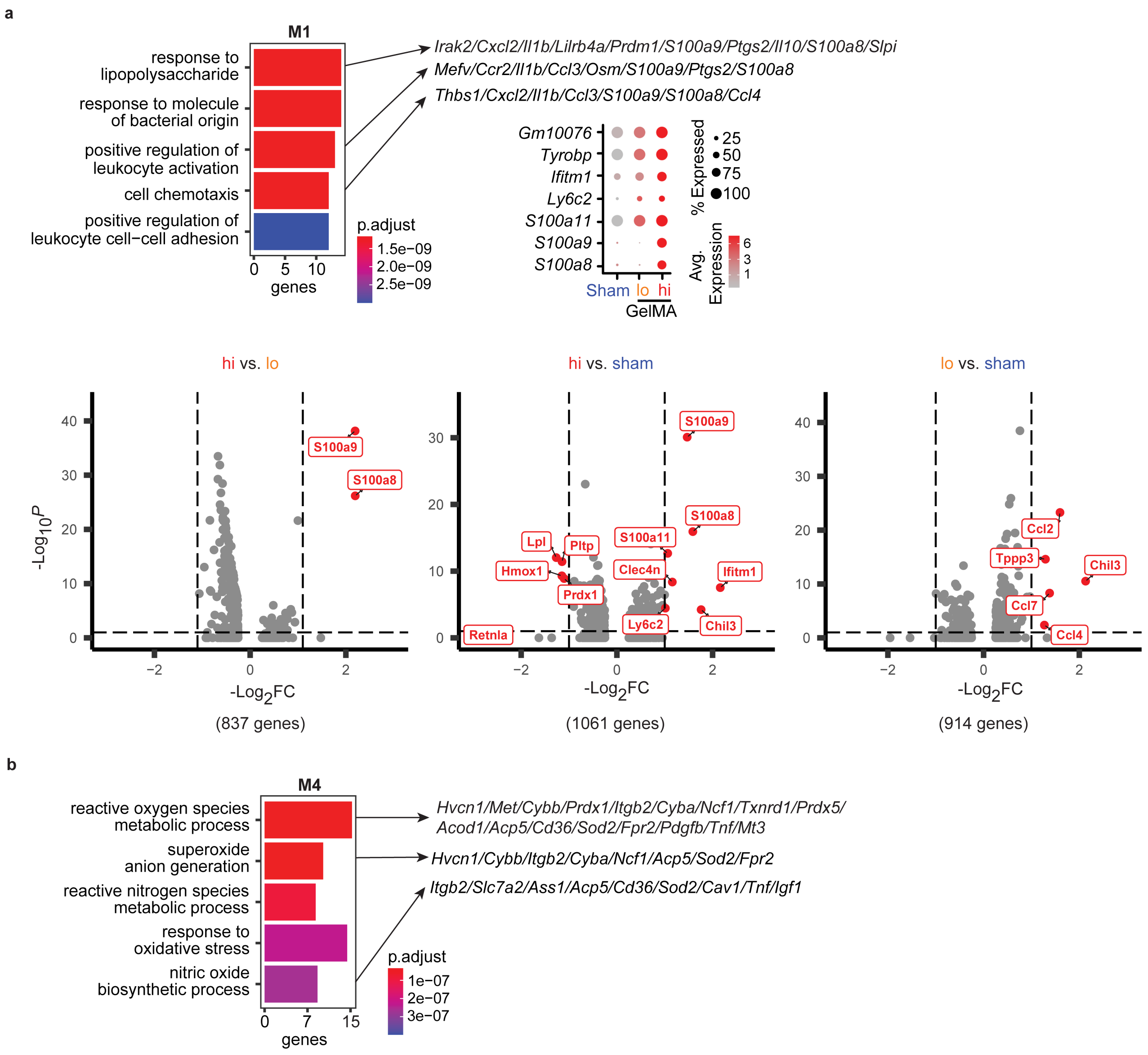

Supplementary Fig. 2. Profiling of Mϕs enriched in hi-GelMA; M1, M4

a M1 (monocyte-Mϕ/pro-inflammatory). Bar plot of enriched GO biological processes terms and the constituent genes within terms. Dot plot of hyper-inflammatory genes across treatments. Dot size corresponds to proportion of cells within the group expressing each gene, and dot color corresponds to the expression level. Volcano plots of the differentially expressed genes across treatments. **b** M4 (pro-inflammatory/catabolic/oxidative). Bar plot of enriched GO biological processes terms and the constituent genes within terms.

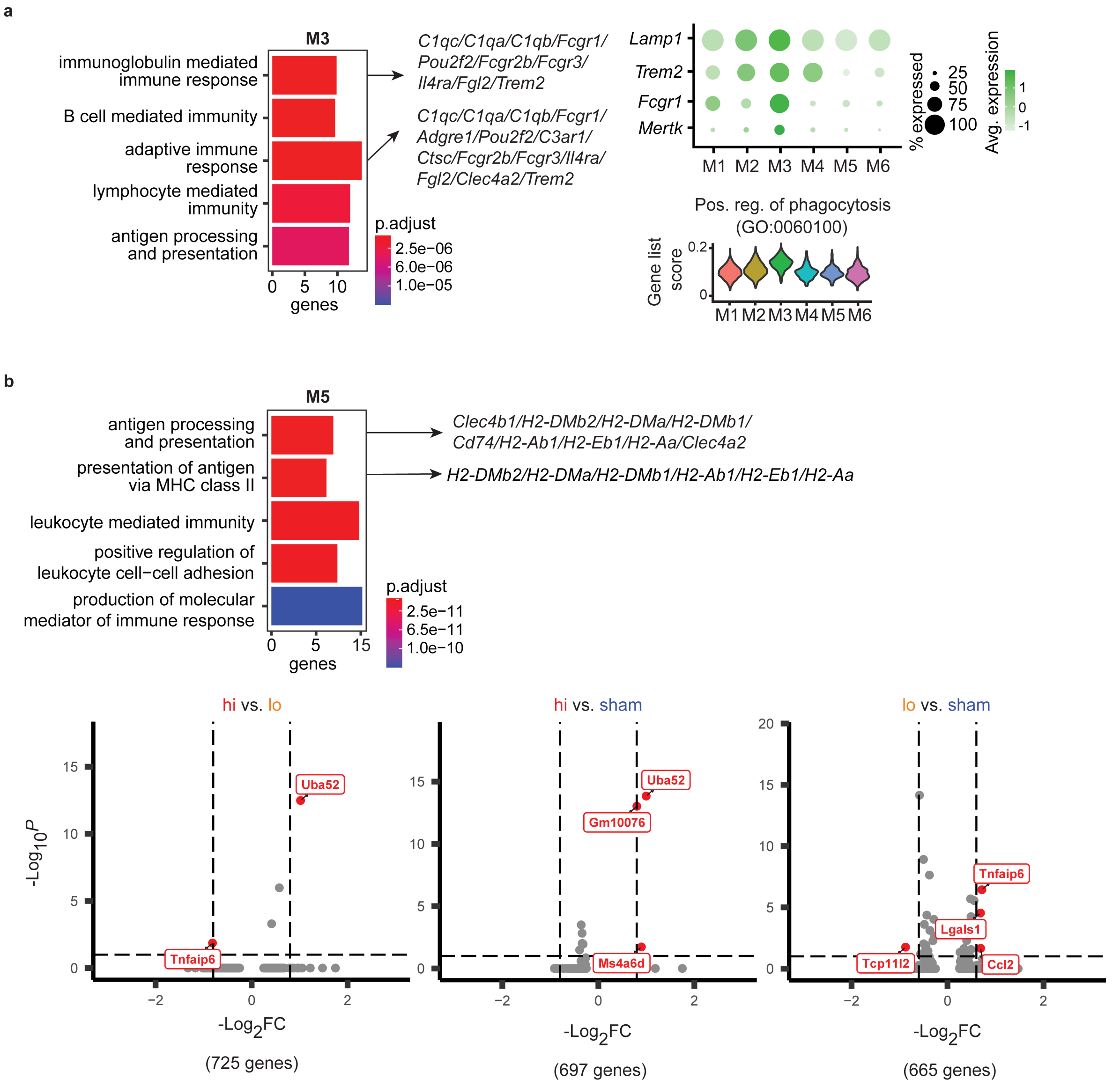

Supplementary Fig. 3. Profiling of Mϕs enriched in lo-GelMA; M3, M5

a M3 (phagocytic/complement activity/Ag processing). Bar plot of enriched GO biological processes terms and the constituent genes within terms. Dot plot of phagocytic genes across Mϕ subpopulations. Violin plot of scores on phagocytic gene list across Mϕ subpopulations. **b** M5 (pro-healing/Ag processing). Bar plot of enriched GO biological processes terms and the constituent genes within terms. Volcano plots of the differentially expressed genes across treatments.

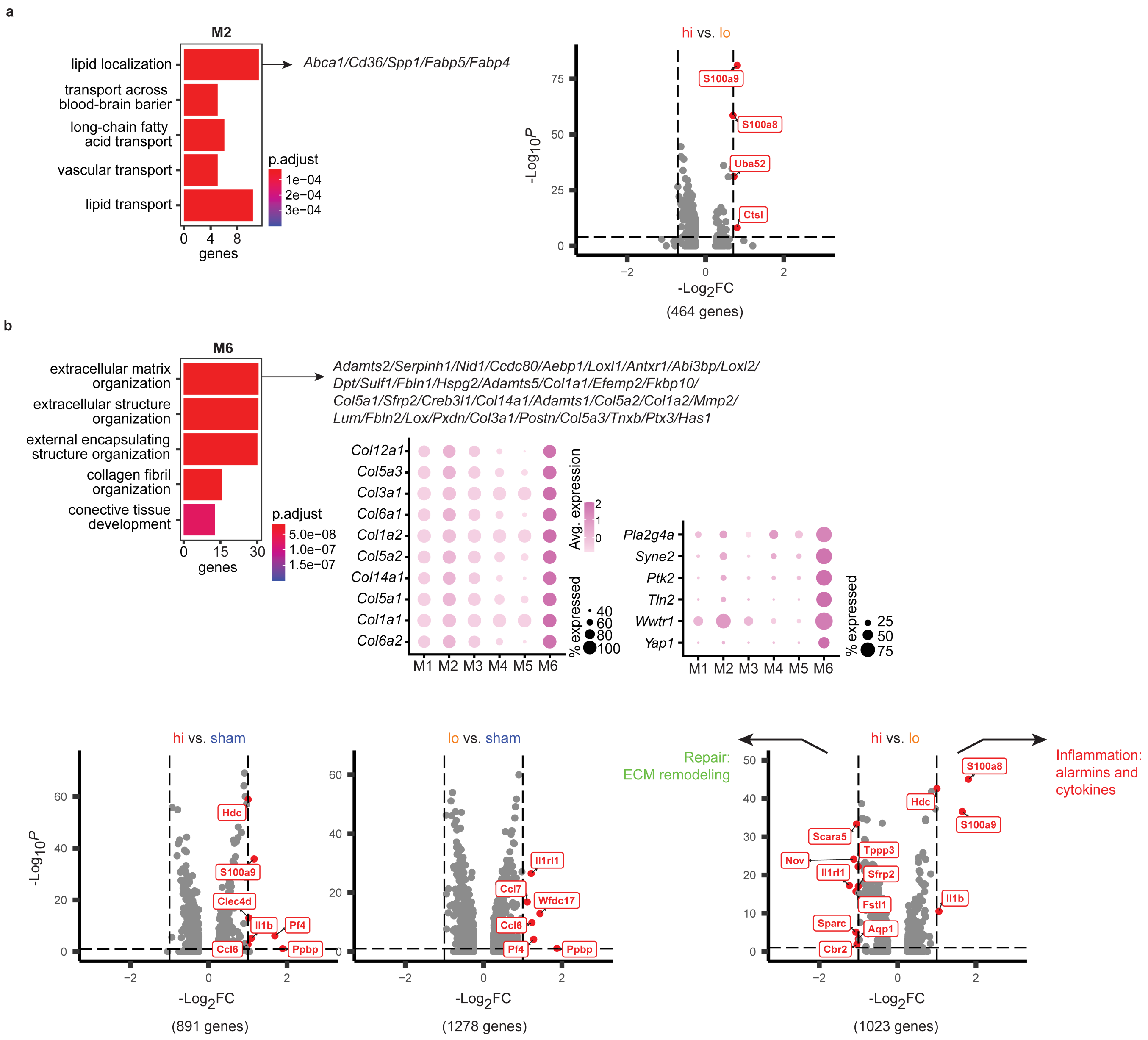

Supplementary Fig. 4. Profiling of Mϕs; M2, M6

a M2 (pro-inflammatory/phagocytic/chemotactic/lipid processing). Bar plot of enriched GO biological processes terms and the constituent genes within terms. Volcano plot of the differentially expressed genes in hi- vs. lo-GelMA. **b** M6 (collagenic/reparative). Bar plot of enriched GO biological processes terms and the constituent genes within terms. Dot plots of collagen genes and mechanotransduction genes across treatments. Dot size corresponds to proportion of cells within the group expressing each gene, and dot color corresponds to the expression level. Volcano plots of the differentially expressed genes across treatments.

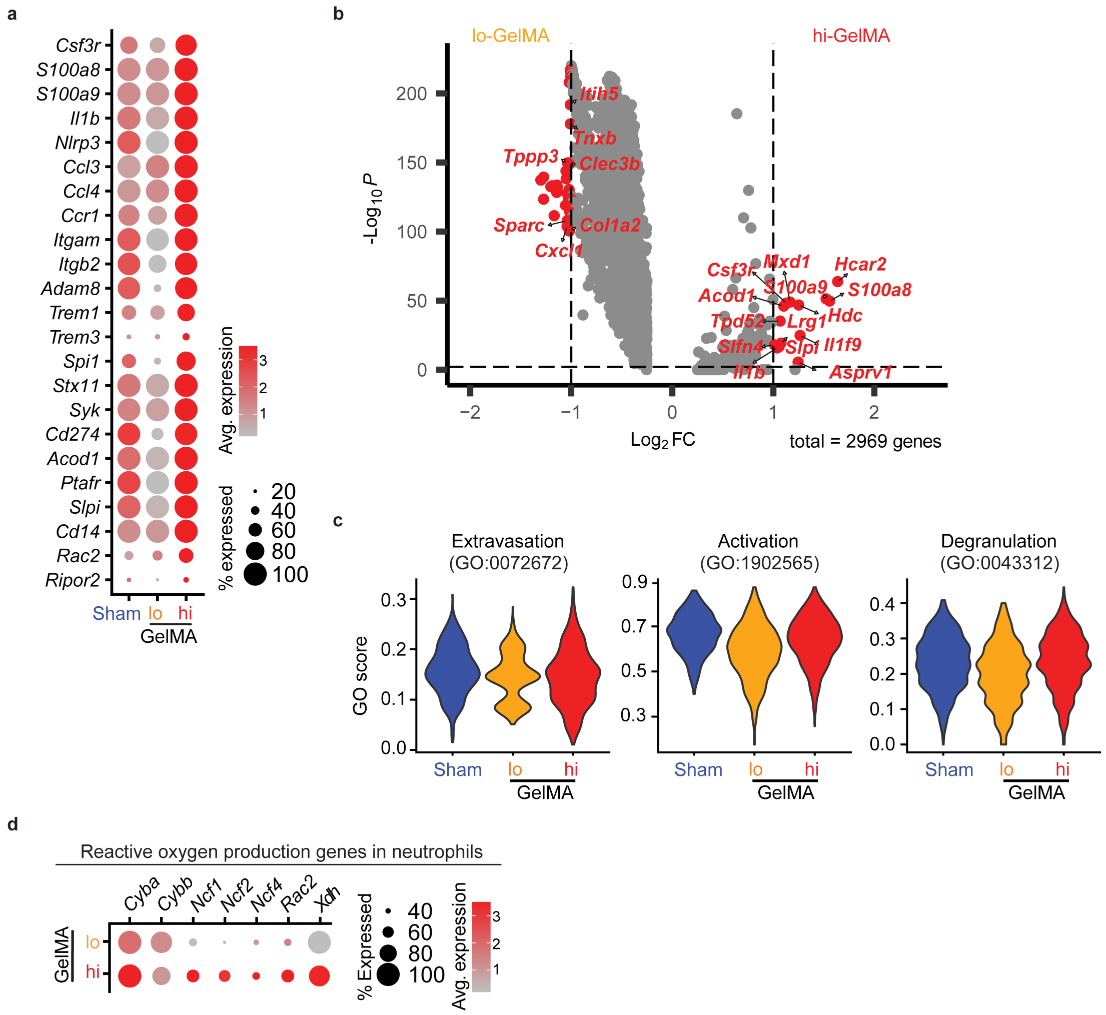

Supplementary Fig. 5. Crosslinking of GelMA hydrogel dressings modulates neutrophil inflammatory activation in wound healing.

a Dot plot of inflammatory genes expressed by neutrophils across different treatments. Dot size corresponds to proportion of cells within the group expressing each transcript, and dot color corresponds to the expression level. **b** Volcano plot of the differentially expressed genes in lo- vs. hi-GelMA. **c** Gene scoring of GO terms related to neutrophil activation. **d** Dot plot of reactive oxygen production genes expressed by neutrophils in lo- vs. hi-GelMA.

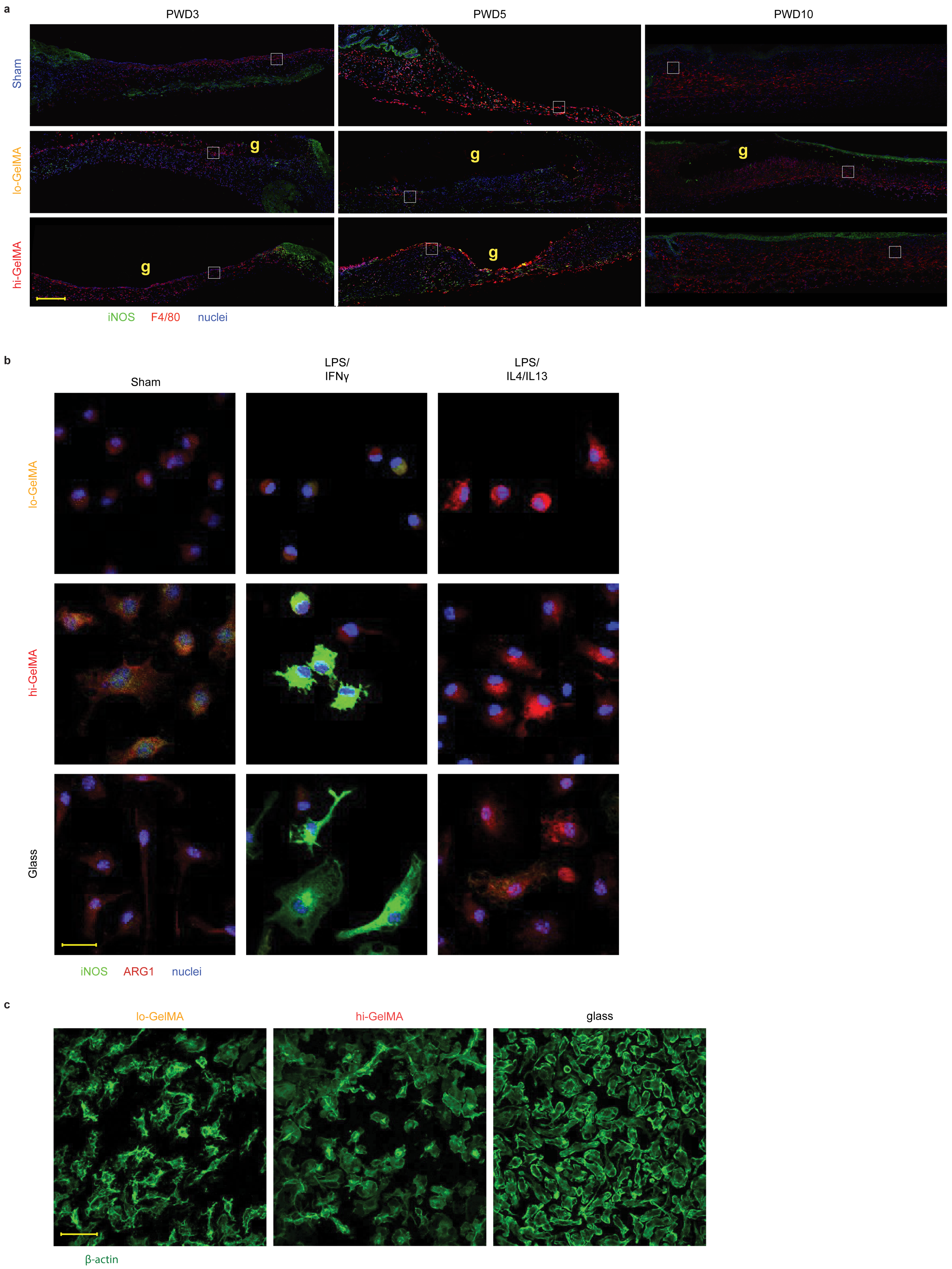

Supplementary Fig. 6. Crosslinking of GelMA hydrogels modulates Mϕ function and wound healing

a Supplementary images for Fig. 2e. Immunohistochemistry of wounds at PWD3, 5 and 10 across treatment groups, stained for Mϕ marker F4/80, and inflammatory marker iNOS. Scale bar: 200 µm. **b** Representative fluorescent images of BMDM cultured on lo- or hi-GelMA, or glass (as in Fig. 2f-g), activated with cytokines IFN-γ or IL-4+IL-13 and stimulated with LPS. Scale bar: 25 µm. **c** BMDMs were seeded on the gels for 24h, then fixed, stained with the actin stain phalloidin, and imaged with laser scanning microscope. Scale bar: 25 µm.

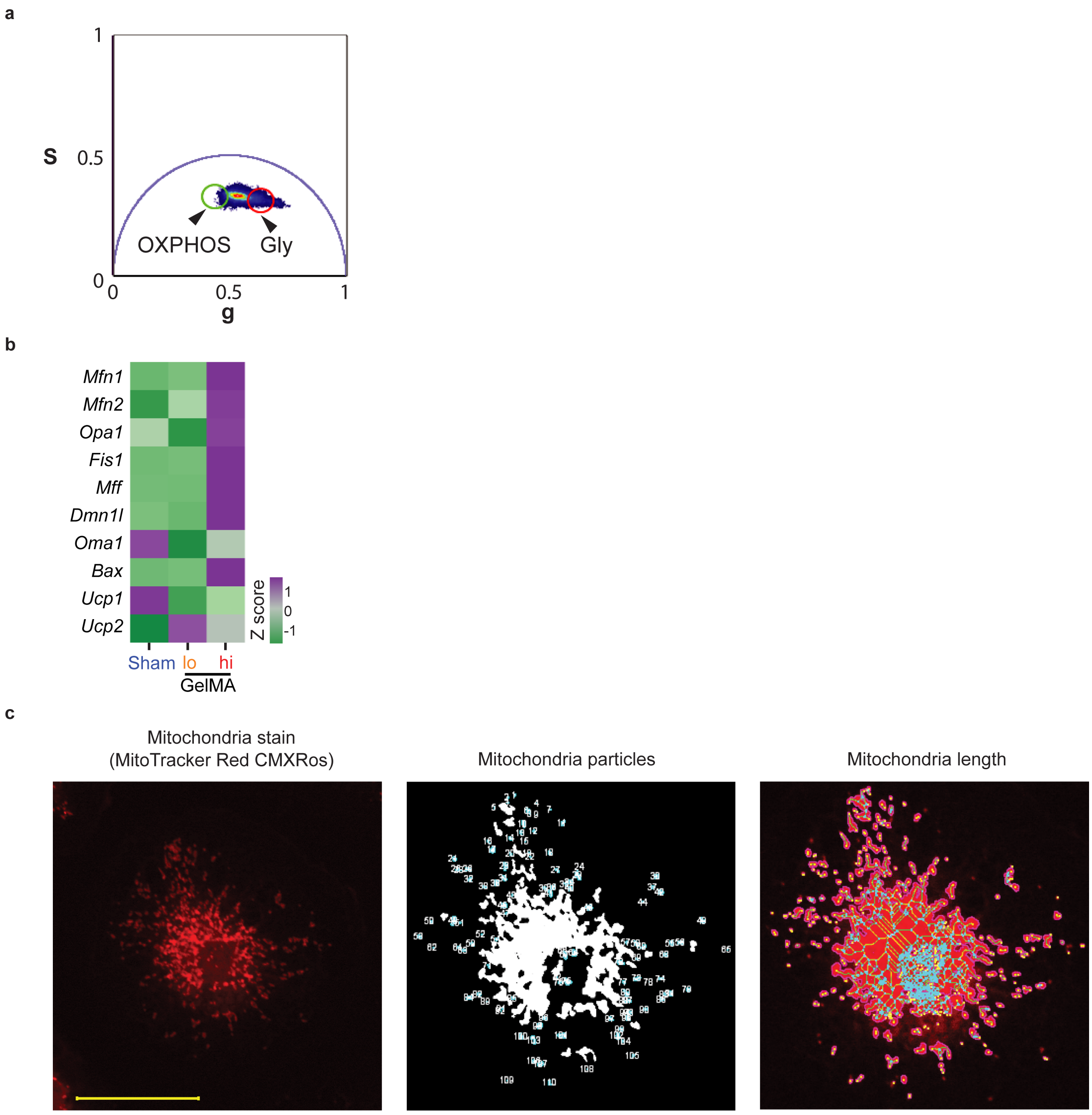

Supplementary Fig. 7. Crosslinking of GelMA substrate modulates mitochondrial dynamics.

a FLIM analysis illustration of the distribution of free and protein-bound NADH in cells, with the 'g' and 's' axes representing the phase and modulation components of fluorescence decay, respectively. The positioning of points on the plot reflects the metabolic state of the cells, differentiating between shorter (free NADH) and longer (bound NADH) fluorescence lifetimes. **b** Heat map of mitochondria dynamics genes between different treatments. **c** Representative images of measurements for Fig. 3i. Laser scanning microscopy images of BMDMs seeded on hi vs. lo-GelMA or glass for 24h followed by LPS stimulation for 16h and stained with MitoTracker Red CMXRos.

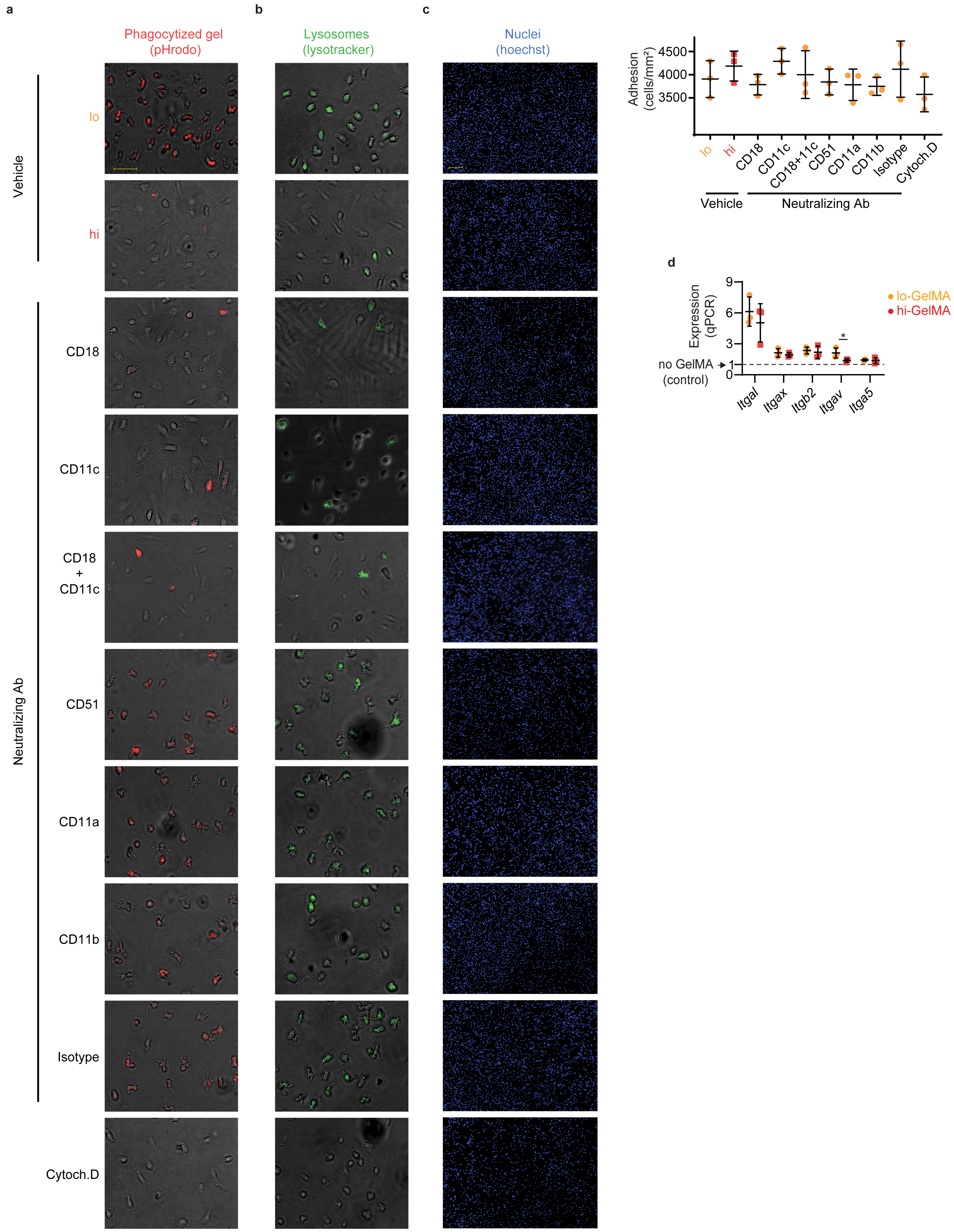
a
b
c
Phagocytized gel
(pHrodo)
Lysosomes
(lysotracker)
Nuclei
(hoechst)
Vehicle
lo
hi
Neutralizing Ab
CD18
CD11c
CD18
+
CD11c
CD51
CD11a
CD11b
Isotype
Cytoch.D
Adhesion
(cells/mm²)
4500
4000
3500
lo
hi
CD18
CD11c
CD18+11c
CD51
CD11a
CD11b
Isotype
Cytoch.D
Vehicle
Neutralizing Ab
d
Expression
(qPCR)
9
6
3
1
0
no GelMA
(control)
*
Itgal
Itgax
Itgb2
Itgav
Itga5
lo-GelMA
hi-GelMA

Supplementary Fig. 8. Differential modulation of macrophage phagocytosis by GelMA crosslinking.

a Supplemental images for plate reader measurements in Fig. 4e. BMDMs seeded on pHrodo-labeled (red) hi- or lo-GelMA for 16h, treated with 10 µg/ml blocking antibodies CD18, CD11c, CD51, CD11a, and CD11b. Compared to 10 µM phagocytosis inhibitor Cytochalasin-D (Cytoch.D) and antibody isotype controls (Isotype). Scale bar: 25 µm. **b** Supplemental images for plate reader measuring in Fig. 4f. Similar as in (**a**), BMDMs seeded on unlabeled gels stained with lysotracker (green). **c** Similar as in (**a-b**), images of BMDMs stained with Hoechst, treated with conditions as in (**a-b**), washed, imaged and measured for adhesion. n=3 (BMDM harvests from individual mice). Scale bar: 150 µm. **d** Integrin gene expression on BMDMs cultured on lo vs. hi-GelMA for 24h, compared to BMDMs cultured without GelMA. n=3 (BMDM harvests from individual mice). p = 0.06 (t-test, two-sided). All data presented as mean ± SD. Source data are provided as a Source Data file.

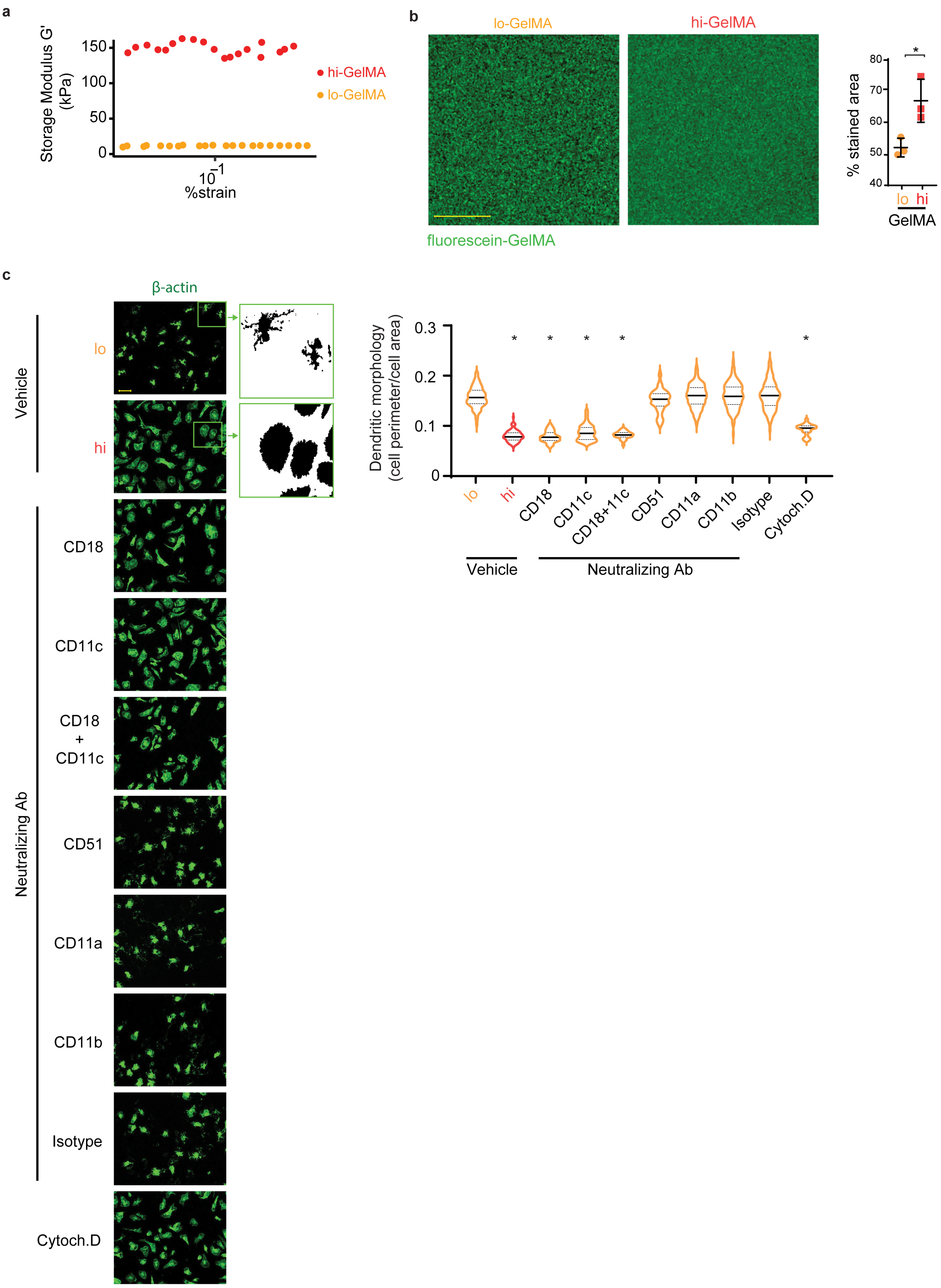

Supplementary Fig. 9 Crosslinking of GelMA substrate modulates stiffness, density and the resulted BMDMs morphology.

a Rheometer measurement of GelMA stiffness was characterized by a parallel plate rheometer. **b** hi and lo-GelMA hydrogels were stained after polymerization with fluorescein NHO-ester, washed and imaged with laser scanning microscope. Quantification measuring the dye coverage as indication of substrate density. n=3. p = 0.02 (t-test, two-sided). Scale bar: 25 µm. **c** BMDMs seeded hi- or lo-GelMA for 16h, treated with 10 µg/ml blocking antibodies CD18, CD11c, CD51, CD11a, and CD11b. Compared to 10 µM phagocytosis inhibitor Cytochalasin-D (Cytoch.D) and antibody isotype controls (Isotype). Then, BMDMs were fixed, stained with the actin stain phalloidin, and imaged with laser scanning microscope. Violin plot showing the dendritic morphology index by dividing cell perimeter to the cell area. n=100 (BMDMs from 3 individual mice). $p < 0.0001$(one-way ANOVA, Tukey's HSD). Scale bar: 25 µm. All data presented as mean ± SD. Source data are provided as a Source Data file.

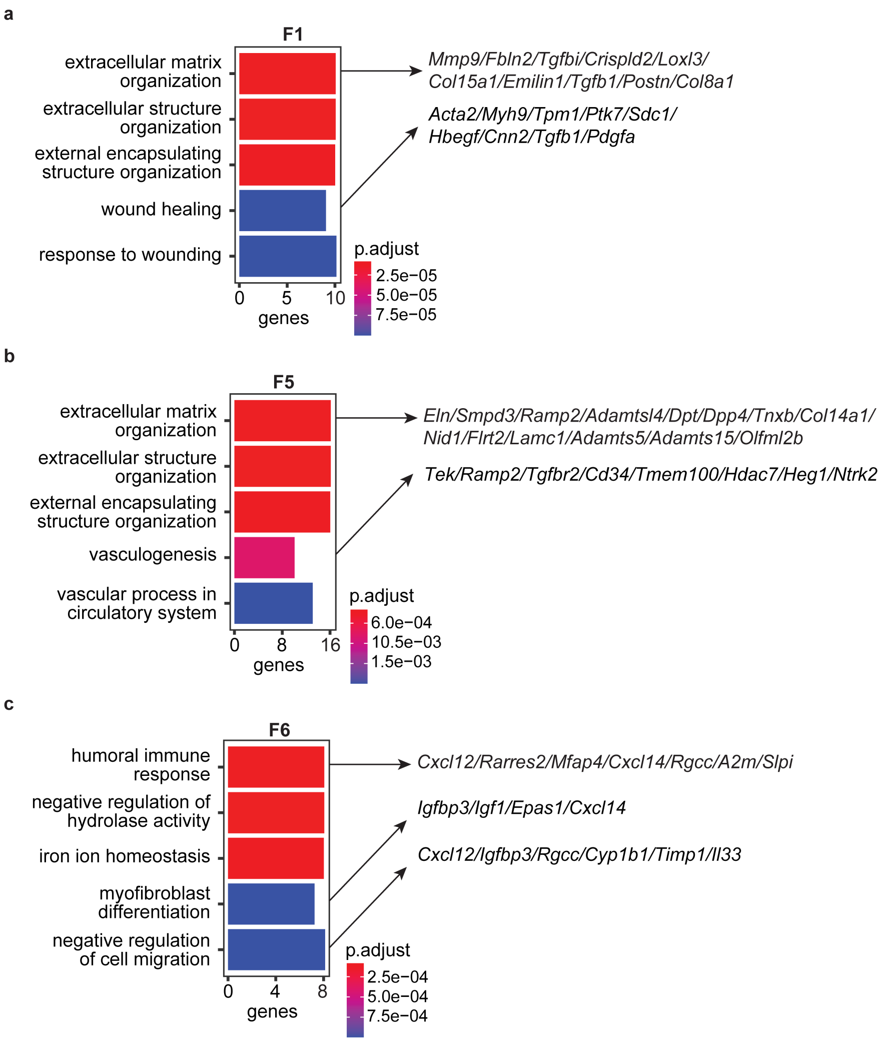

Supplementary Fig. 10. Profiling of fibroblasts enriched in hi-GelMA; F1, F5 and F6.

a F1 (myofibroblast). Bar plot of enriched GO biological processes terms and the constituent genes within terms. **b** F5 (angiogenic). Bar plot of enriched GO biological processes terms and the constituent genes within terms. **c** F6 (myofibroblasts/ proliferating). Bar plot of enriched GO biological processes terms and the constituent genes within terms.

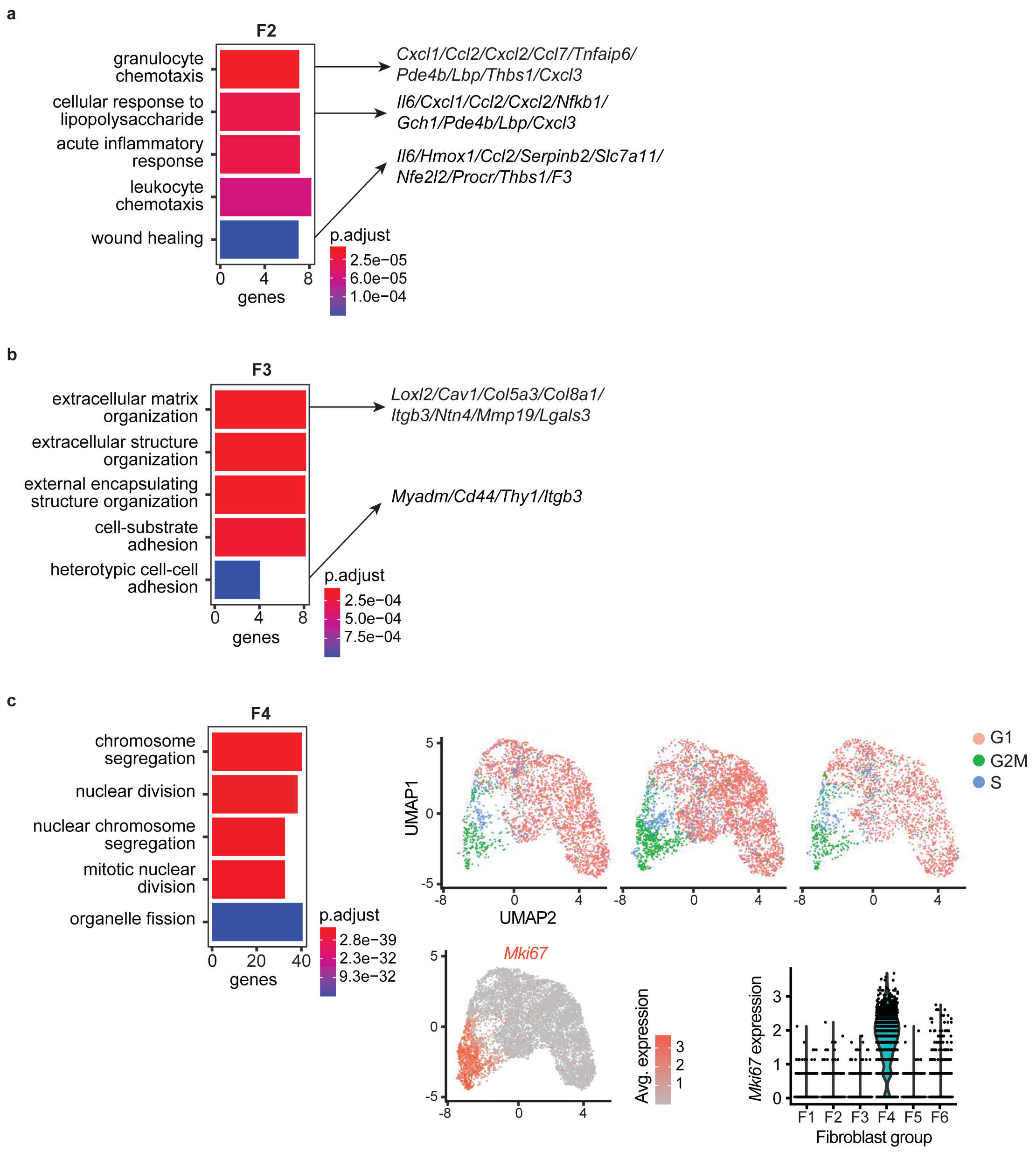

Supplementary Fig. 11. Profiling of fibroblasts enriched in lo-GelMA; F2, F3 and F4.

a F2 (chemotactic). Bar plot of enriched GO biological processes terms and the constituent genes within terms. **b** F3 (collagen crosslinking). Bar plot of enriched GO biological processes terms and the constituent genes within terms. **c** F4 (proliferating). Bar plot of enriched GO biological processes terms and the constituent genes within terms. Feature plot with gene scoring of fibroblast cell cycling phases red: G1, green: G2M (G to mitosis transition) and blue: S (synthesis). *Mki67* expression in fibroblasts on feature plot and violin plot across subpopulations.

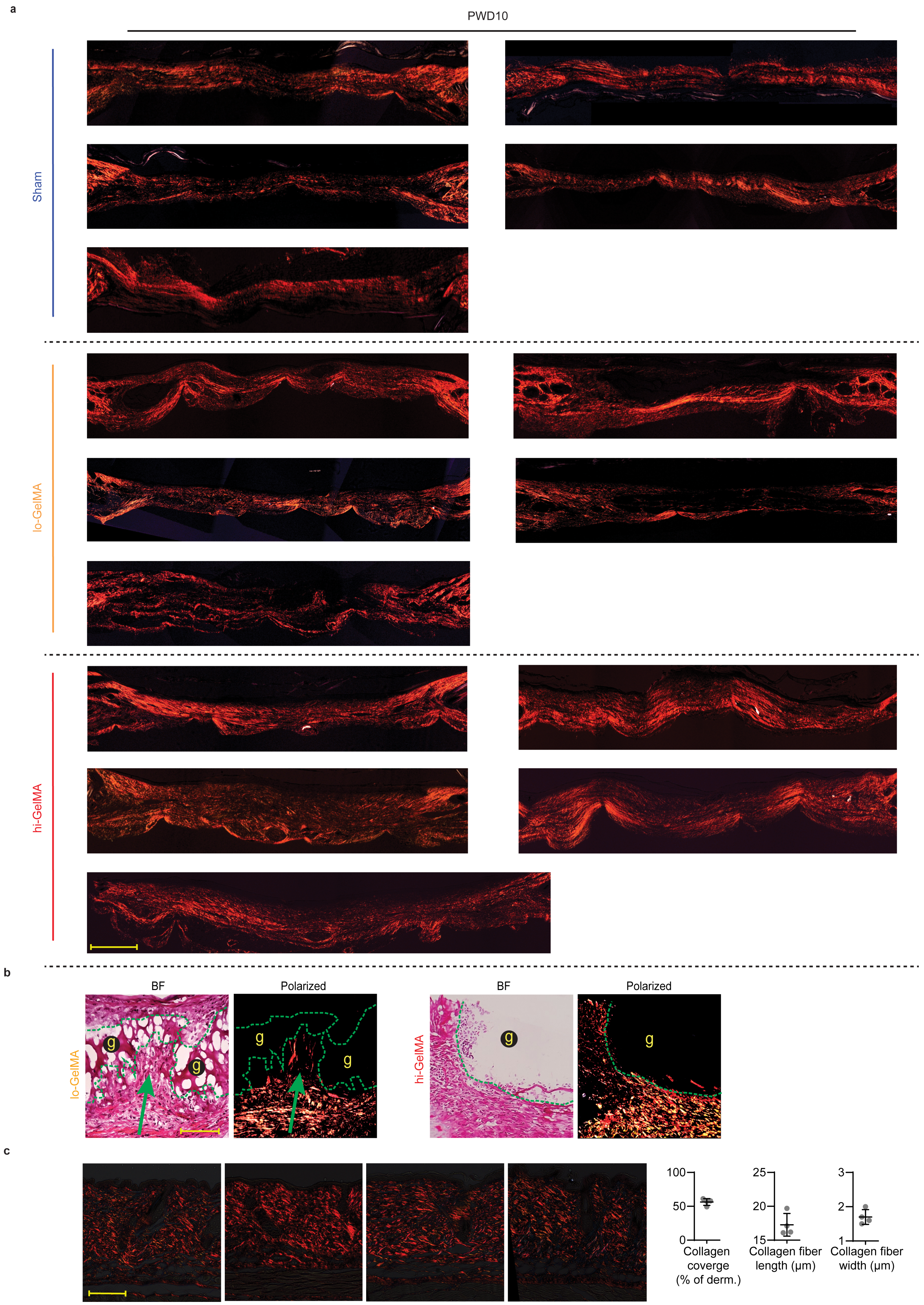

a
PWD10
Sham
lo-GelMA
hi-GelMA
b
BF
Polarized
BF
Polarized
lo-GelMA
hi-GelMA
g
c
100
50
0
Collagen coverge (% of derm.)
25
20
15
Collagen fiber length (μm)
3
2
1
Collagen fiber width (μm)

Supplementary Fig. 12. Additional PSR staining of collagen in PWD10 wound sections

a Images of PSR staining of collagen in PWD10 wound sections showing sham wounds along with GelMA treated wounds with enhances fibrotic features in hi-GelMA vs. lo-GelMA including enhanced collagenous dermis width, collagen coverage of dermis, and collagen fiber length and width. Scale bar: 200 µm. **b** Deposition of mature collagen bundles only in lo-GelMA and absence in hi-GelMA; H&E images are shown adjacent for reference. Green dashed line: GelMA-dermis interphase. Green arrows: matured collagen bundles. g: GelMA. Scale bar: 25 µm. **c** Images of PSR staining of collagen in normal skin sections (non-wounded), presenting overall lower quantities of fibrotic features compared to Fig. 4f. n=4 (mice). Scale bar: 50 µm. Source data are provided as a Source Data file.

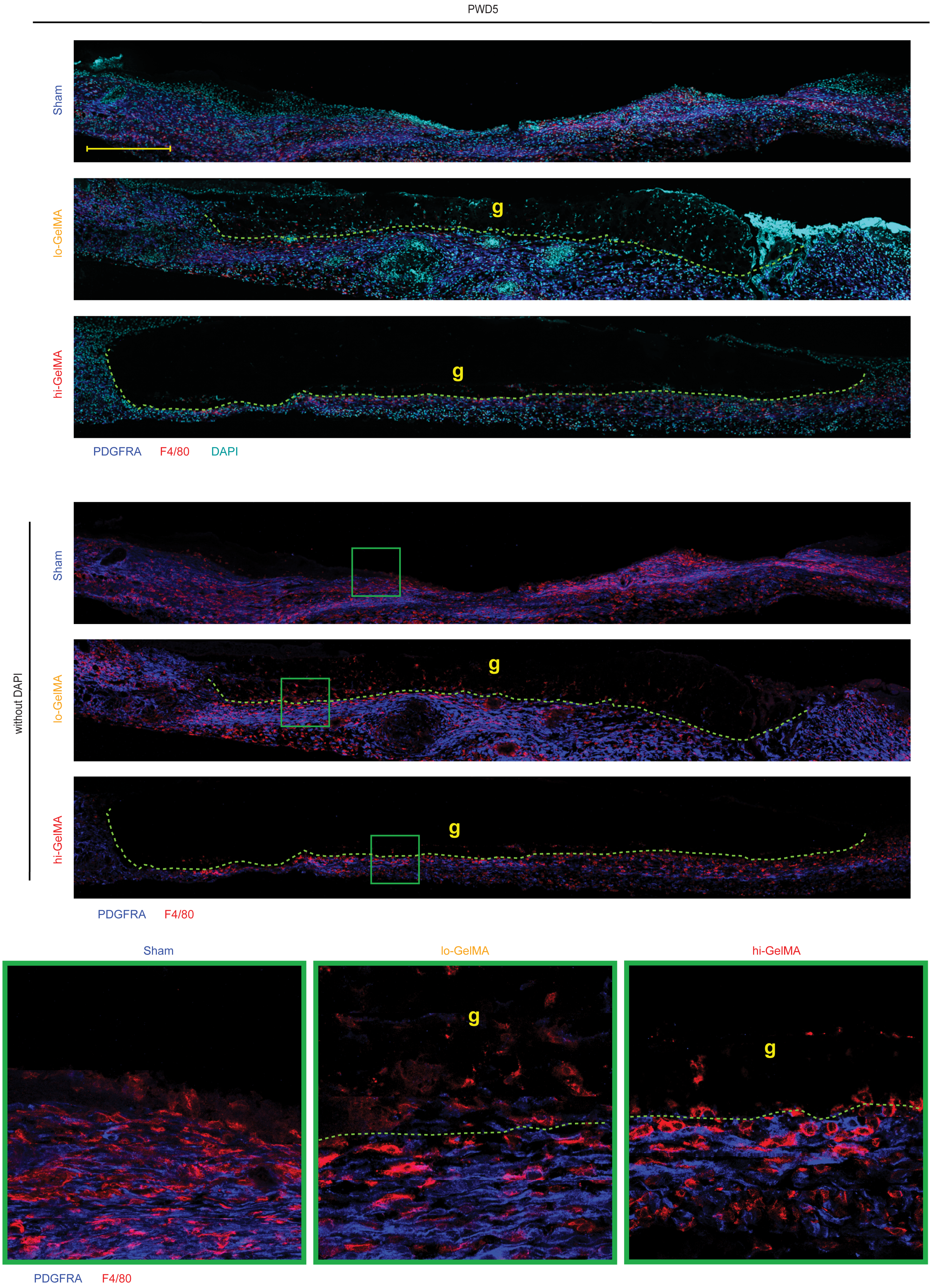
PWD5
Sham
lo-GelMA
hi-GelMA
g
g
PDGFRA
F4/80
DAPI
without DAPI
Sham
lo-GelMA
hi-GelMA
g
g
PDGFRA
F4/80
Sham
lo-GelMA
hi-GelMA
g
g
PDGFRA
F4/80

Supplementary Fig. 13. Macrophages and fibroblasts colocalization throughout wound healing at PWD3, 5 and 10

Immunohistochemistry analysis of wounds at PWD5 across treatment groups, stained for F4/80 (Mϕ marker) and PDGFRA (fibroblast marker). With DAPI stain (top panel), without DAPI (central panel). Green boxes indicate regions that have been shown at higher magnification (bottom panel). Green dashed line: GelMA-dermis border. g: GelMA. Scale bar: 200 μm.
