# Supplementary material for: Hydrogel crosslinking modulates macrophages, fibroblasts, and their communication, during wound healing
Source: Nat Commun. 2024 Aug 9;15:6820. doi: 10.1038/s41467-024-50072-y (PMC11315930; doi:10.1038/s41467-024-50072-y)
Supplement: Supplementary file 3 — Reporting Summary [file 41467_2024_50072_MOESM3_ESM.pdf]

nature portfolio

Corresponding author(s): NCOMMS-23-40286B

Last updated by author(s): May 11, 2024

Reporting Summary

Nature Portfolio wishes to improve the reproducibility of the work that we publish. This form provides structure for consistency and transparency in reporting. For further information on Nature Portfolio policies, see our Editorial Policies and the Editorial Policy Checklist.

Statistics

For all statistical analyses, confirm that the following items are present in the figure legend, table legend, main text, or Methods section.

n/a	Confirmed	
☐	☒	The exact sample size (*n*) for each experimental group/condition, given as a discrete number and unit of measurement
☐	☒	A statement on whether measurements were taken from distinct samples or whether the same sample was measured repeatedly
☐	☒	The statistical test(s) used AND whether they are one- or two-sided<br>*Only common tests should be described solely by name; describe more complex techniques in the Methods section.*
☐	☒	A description of all covariates tested
☐	☒	A description of any assumptions or corrections, such as tests of normality and adjustment for multiple comparisons
☐	☒	A full description of the statistical parameters including central tendency (e.g. means) or other basic estimates (e.g. regression coefficient) AND variation (e.g. standard deviation) or associated estimates of uncertainty (e.g. confidence intervals)
☐	☒	For null hypothesis testing, the test statistic (e.g. *F*, *t*, *r*) with confidence intervals, effect sizes, degrees of freedom and *P* value noted<br>*Give P values as exact values whenever suitable.*
☒	☐	For Bayesian analysis, information on the choice of priors and Markov chain Monte Carlo settings
☒	☐	For hierarchical and complex designs, identification of the appropriate level for tests and full reporting of outcomes
☒	☐	Estimates of effect sizes (e.g. Cohen's *d*, Pearson's *r*), indicating how they were calculated

Our web collection on statistics for biologists contains articles on many of the points above.

Software and code

Policy information about availability of computer code

Data collection	Olympus FV3000 laser scanning microscope: Utilized for imaging cellular and tissue samples. V4.5.<br>In Vivo Imaging System (IVIS): Used for live animal imaging to monitor wound healing. V4.7.<br>Zeiss LSM 880 microscope: Employed for acquiring fluorescence lifetime images. V: ZEN 3.0.<br>TA instruments' DHR3 instrument: Used for rheological measurements to characterize GelMA stiffness. TRIOS 4.1.3.
Data analysis	Fiji-ImageJ: Utilized for dermis width measurement, mitochondria and cytoskeleton morphological analysis. (v 2.1.0).<br>Seurat R package: Employed for cell clustering and marker gene identification in single-cell RNA sequencing data. (v 4.0.1).<br>clusterProfiler: Used for Gene Ontology (GO) analysis to identify putative signaling pathways. (v 4.0.5).<br>Monocle 3: Applied for cell lineage and pseudotime inference in single-cell RNA sequencing data. (v 2.18.0).<br>SimFCS: Used for FLIM (Fluorescence Lifetime Imaging Microscopy) image acquisition and processing.<br>CellChat: Employed for cell-cell interaction network analysis in single-cell RNA sequencing data. (v 1.1.0).<br>Ucell: Used for single-cell gene signature scoring in single-cell RNA sequencing data.<br>Mitochondrial Network Analysis (MiNA) toolset: Employed for analyzing the morphology of mitochondria.

For manuscripts utilizing custom algorithms or software that are central to the research but not yet described in published literature, software must be made available to editors and reviewers. We strongly encourage code deposition in a community repository (e.g. GitHub). See the Nature Portfolio guidelines for submitting code & software for further information.

Data

Policy information about availability of data

All manuscripts must include a data availability statement. This statement should provide the following information, where applicable:

- Accession codes, unique identifiers, or web links for publicly available datasets
- A description of any restrictions on data availability
- For clinical datasets or third party data, please ensure that the statement adheres to our policy

Materials and protocols generated in this study will be made available upon request and may require a material transfer agreement. Sequencing data will be deposited according to the guidelines.

Research involving human participants, their data, or biological material

Policy information about studies with human participants or human data. See also policy information about sex, gender (identity/presentation), and sexual orientation and race, ethnicity and racism.

Reporting on sex and gender	NA
Reporting on race, ethnicity, or other socially relevant groupings	NA
Population characteristics	NA
Recruitment	NA
Ethics oversight	NA

Note that full information on the approval of the study protocol must also be provided in the manuscript.

Field-specific reporting

Please select the one below that is the best fit for your research. If you are not sure, read the appropriate sections before making your selection.

☒ Life sciences ☐ Behavioural & social sciences ☐ Ecological, evolutionary & environmental sciences

For a reference copy of the document with all sections, see nature.com/documents/nr-reporting-summary-flat.pdf

Life sciences study design

All studies must disclose on these points even when the disclosure is negative.

Sample size	Sample sizes for murine studies were determined according to or exceeding standards in the field. Specifically, p50 C57BL/6J mice were utilized in the wounding studies. For bone marrow-derived macrophages (BMDM) culture, femur and tibia bones were harvested from C57BL/6J mice.
Data exclusions	Data were carefully curated to ensure quality. For example, in sequencing data analysis, cells with greater than 5% mitochondrial DNA content, a total number of > 7,500 and/or <200 genes expressed were excluded from further analysis. The rationale behind this exclusion is to ensure data quality and reliability. An experiment of scRNAseq on wound samples was determined disqualified and unrelibable due to having ~20% mitochondrial DNA. This experiment was excluded.
Replication	Experimental assays were performed in multiple replicates to ensure reproducibility. For example, wounding studies were monitored daily for signs of infection/healing, and at multiple time-points post-wounding, mice were sacrificed for further analysis. Furthermore, ELISA assays, immunofluorescence staining, and other molecular analyses were performed in accordance with standard protocols, and multiple biological replicates were used.
Randomization	Mice were allocated randomly into experimental groups based on the wound treatment they received,
Blinding	Given the rigorous and standardized methods employed, such as UV crosslinking times for GelMA and the use of established biochemical assays, blinding was not deemed necessary or relevant for this study.

Reporting for specific materials, systems and methods

We require information from authors about some types of materials, experimental systems and methods used in many studies. Here, indicate whether each material, system or method listed is relevant to your study. If you are not sure if a list item applies to your research, read the appropriate section before selecting a response.

Materials & experimental systems

n/a	Involved in the study	
☐	☒	Antibodies
☐	☒	Eukaryotic cell lines
☒	☐	Palaeontology and archaeology
☐	☒	Animals and other organisms
☒	☐	Clinical data
☒	☐	Dual use research of concern
☒	☐	Plants

Methods

n/a	Involved in the study	
☒	☐	ChIP-seq
☒	☐	Flow cytometry
☒	☐	MRI-based neuroimaging

Antibodies

Antibodies used	F4/80, Supplier: Thermo Fisher, Catalog Number: MF48000 BM8; Arginase, Supplier: Abcam, Catalog Number: 60176; iNOS, Supplier: Abcam, Catalog Number: 15323; α-SMA, Supplier: Abcam, Catalog Number: 5694; PDGFR-α, Supplier: Abcam, Catalog Number: AF1062, GAPDH, 1:2000 (WB), BioLegend (607902).
Validation	Manufacturer's validation

Eukaryotic cell lines

Policy information about cell lines and Sex and Gender in Research

Cell line source(s)	The primary cell lines used in this study were bone marrow-derived macrophages (BMDMs) harvested from C57BL/6J mice. All primary cell lines were sourced from female mice.
Authentication	BMDMs produced according to standardized protocols and authentication by microscopy and functional assays e.g. ELISA, WB.
Mycoplasma contamination	Mycoplasma contamination tested routinely but not on every cell culture batch.
Commonly misidentified lines (See ICLAC register)	NA

Animals and other research organisms

Policy information about studies involving animals; ARRIVE guidelines recommended for reporting animal research, and Sex and Gender in Research

Laboratory animals	Female C57BL/6 mice. Age: for BMDM: 6-12 weeks, for wounds: p50.
Wild animals	NA
Reporting on sex	Only female C57BL/6 mice used.
Field-collected samples	NA
Ethics oversight	University of California, Irvine Institutional Animal Care and Use Committee

Note that full information on the approval of the study protocol must also be provided in the manuscript.

Plants

Seed stocks	NA
Novel plant genotypes	NA
Authentication	NA
